# Alterations in gut microbiota and plasma metabolites in pulmonary arterial hypertension secondary to congenital left-to-right shunt heart disease: potential mechanisms and biomarkers

**DOI:** 10.3389/fgene.2026.1699787

**Published:** 2026-02-06

**Authors:** Diwen Li, Tianli Zhao, Xueyang Gong, Yiliya Ahemaiti, Luyao Wei, Yuyang Huang, Shijun Hu

**Affiliations:** 1 Department of Cardiovascular Surgery, The Second Xiangya Hospital, Central South University, Changsha, China; 2 Clinical Center for Gene Diagnosis and Therapy, The Second Xiangya Hospital of Central South University, Changsha, Hunan, China

**Keywords:** congenital heart disease, gut microbiota, microbiome-host interaction, plasma metabolomics, pulmonary arterial hypertension

## Abstract

**Introduction:**

Pulmonary arterial hypertension (PAH) secondary to congenital left-to-right shunt heart disease (CL-RSHD) is a life-threatening complication with unclear microbial and metabolic mechanisms. This study investigated gut microbiota and plasma metabolic alterations in CL-RSHD-associated PAH to identify biomarkers and mechanistic pathways.

**Methods:**

This cross-sectional study included 86 participants: healthy controls (HC, *n* = 13), CL-RSHD (*n* = 46), and CL-RSHD + PAH (*n* = 27). Gut microbiota was analyzed using 16S rRNA gene sequencing of the V3–V4 region on 41 fecal samples (HC, *n* = 9; CL-RSHD, *n* = 15; and CL-RSHD + PAH, *n* = 17). Untargeted plasma metabolomics was analyzed on all 86 plasma samples. Microbial diversity, differential taxa (DESeq2), metabolic pathways (OPLS-DA, KEGG), and biomarker potential (ROC curves) were assessed. Dynamic correlations linked microbiota-metabolite interactions.

**Results:**

CL-RSHD + PAH patients showed preserved α/β-diversity but distinct taxonomic shifts: enriched *Lachnoclostridium phocaeense* (Firmicutes) and reduced SCFA-producing Anaerostipes. Metabolomics revealed dysregulated steroid biosynthesis, cortisol metabolism, and oxidative stress pathways. Key metabolites, including elevated 5-hydroxymethylcytidine (5-hmC) and γ-L-glutamyl-L-cysteine, and reduced histidine intermediate D-E1IG3P, correlated with PAH severity. Strong microbiota-metabolite interactions (e.g., *Lactonifactor*-D-E1IG3P, r = 0.82, *P* < 0.01) suggested a disrupted vascular remodeling axis. Metabolites like ADP-glucose (AUC = 0.94) and 3-phenylpropyl glucosinolate (AUC = 0.92) showed high diagnostic accuracy.

**Conclusion:**

CL-RSHD-associated PAH involves gut microbial restructuring and metabolic reprogramming linked to immune-inflammatory activation and oxidative stress. The Firmicutes-histidine metabolism axis emerges as a therapeutic target. Despite limitations, this study provides foundational insights into microbial-metabolic drivers of PAH, highlighting novel biomarkers for early diagnosis and intervention.

## 1 Introduction

Congenital heart disease (CHD) is a congenital malformation resulting from abnormal cardiovascular development during fetal life (van der Linde et al., 2011). Currently, the number of adults with CHD is steadily increasing. Pulmonary arterial hypertension (PAH) in CHD usually results from intracardiac or extracardiac shunts that create pressure or volume overload in the pulmonary circulation. This chronic overload induces shear stress, endothelial injury, and detrimental pulmonary vascular remodeling. While the precise prevalence of PAH is unclear, it is estimated that approximately 10% of adults with CHD develop PAH, which significantly affects their quality of life and prognosis (Engelfriet et al., 2007).

The gut microbiome actively participates in digestion, vitamin production, pathogen defense, and immune system regulation (Martin-Gallausiaux et al., 2021; Blaut, 2015). Its composition tends to stabilize between the first and second year of life, depending on diet, physical activity, and overall health (Quigley, 2013). Additionally, metabolites produced by the microbiome affect the function of the gut barrier, thereby influencing human health and the development of various diseases. Increasing evidence shows that compared to healthy individuals, PAH patients exhibit significant changes in their gut microbiome composition (Kim et al., 2020; Moutsoglou et al., 2023), with notable increases or decreases in certain microbial metabolites. Researchers have proposed that classic gut microbiome metabolites, such as TMAO and SCFAs, could serve as biomarkers in the progression of PAH (Moutsoglou et al., 2023; Yang et al., 2022). However, it remains unclear whether the gut microbiome system also undergoes changes in the progression of PAH associated with CHD in adults.

PAH is not only a pulmonary vascular disease but also a severe metabolic disorder. Recently, increasing research has indicated that PAH is considered a systemic disease associated with metabolic dysfunction (Pietra et al., 2004). Growing evidence suggests that PAH patients exhibit multiple metabolic abnormalities (Lewis et al., 2016). For instance, Zhao et al. found that disrupted glycolysis and increased fatty acid metabolism can directly regulate pathological vascular remodeling in advanced PAH (Zhao et al., 2014). Metabolomics can simultaneously report hundreds of metabolites, providing a comprehensive analysis of changes in endogenous and exogenous compounds and systematically showcasing the final metabolic products resulting from gene and protein alterations during disease progression (DeBerardinis and Keshari, 2022). However, metabolomic studies in adult patients with CHD-associated PAH are still scarce.

Therefore, to gain a deeper understanding of the changes in gut microbiome and plasma metabolites in adult patients with CHD-induced PAH, we conducted a clinical study involving healthy individuals, patients with congenital left-to-right shunt heart disease (CL-RSHD), and patients with CL-RSHD leading to PAH (CL-RSHD + PAH). We integrated data from gut microbiome sequencing and plasma metabolomics. This study aims to provide new insights into disease mechanisms and potential therapeutic interventions by combining comprehensive approaches of gut microbiome sequencing and metabolomics analysis, and to identify new biomarkers related to disease progression.

## 2 Methods

### 2.1 Study population

**FIGURE 1:**
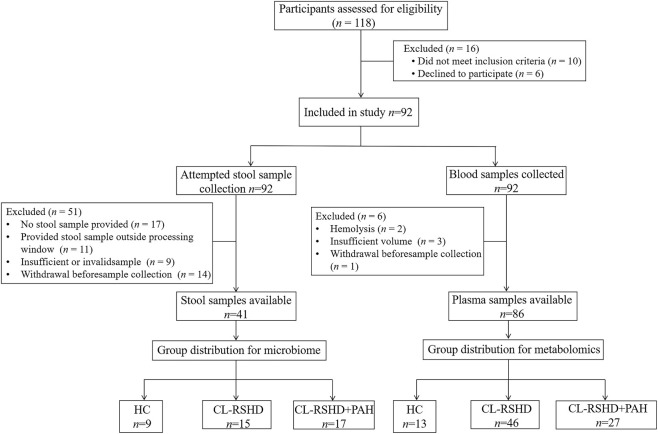
Flowchart of participant recruitment and sample grouping. The flowchart outlines the selection process and sample distribution for the study. Starting from 118 screened participants, 92 were enrolled. (Left) Stool samples: 41 samples remained for microbiome analysis after exclusions, divided into HC (*n*=9), CL-RSHD (*n*=15), and CL-RSHD+PAH (*n*=17). (Right) Plasma samples: 86 samples were available for metabolomics analysis after exclusions, divided into HC (*n*=13), CL-RSHD (*n*=46), and CL-RSHD+PAH (*n*=27). HC, healthy controls; CL-RSHD, congenital left-to-right shunt heart disease; PAH, pulmonary arterial hypertension.

From 01/11/2022 to 01/10/2023, patients diagnosed with CL-RSHD and CL-RSHD + PAH (moderate to severe) at the Second Xiangya Hospital of Central South University were included in our study (Figure 1). Healthy controls were recruited from the relatives of the aforementioned subjects. The data collection and analysis for this study were completed in October 2023. This study did not involve participants under the age of 18. Ethical approval was obtained from the Ethics Committee of the Second Xiangya Hospital of Central South University. This study adheres to the principles of the Declaration of Helsinki. All participants provided written informed consent specifying participation in the study and publication of anonymized data, with ethics approval explicitly covering both aspects.

For our gut microbiome analysis, we excluded participants based on the following criteria: recent use of antibiotics (within the last 1–3 months), other medications affecting the gut microbiome (such as probiotics, prebiotics, laxatives, proton pump inhibitors, immunosuppressants, and chemotherapy drugs), acute or chronic gastrointestinal diseases (such as inflammatory bowel disease, irritable bowel syndrome, diarrhea, constipation), recent gastrointestinal surgery, chronic diseases unrelated to the study (such as diabetes, cirrhosis, heart failure), extreme dietary habits (such as strict vegan or carnivore diets) or recent changes in diet, pregnancy or breastfeeding, and other influencing factors (such as smoking, excessive alcohol consumption, illicit drug use), as well as a personal or family history of hereditary gastrointestinal diseases.

While we recognize that detailed dietary patterns and lifestyle factors (e.g., specific nutrient intake, sleep quality, physical activity metrics) are potential confounders of gut microbiota and metabolism, they were not quantitatively assessed in the present exploratory study due to its cross-sectional design and focus on clinical phenotyping. However, to mitigate their impact, we applied stringent exclusion criteria (as detailed above) that eliminated participants with extreme dietary habits (e.g., strict veganism, carnivore diets), recent major dietary changes, smoking, excessive alcohol consumption, and illicit drug use. This approach was intended to create a more homogeneous cohort regarding major lifestyle confounders.

In addition to the general exclusion criteria, we applied specific exclusion criteria for participants with CL-RSHD and CL-RSHD + PAH. These included excluding individuals with other cardiovascular diseases not related to CL-RSHD or CL-RSHD + PAH, recent cardiac interventions or surgeries specific to CHD or PAH treatment, and any other medical conditions that might interfere with the study’s focus on gut microbiome differences in CHD and PAH patients. Inclusion criteria for CL-RSHD patients were based on echocardiographic diagnosis. For PAH caused by CL-RSHD patients, inclusion criteria were based on right heart catheterization results, with classical definitions of mean pulmonary artery pressure (mPAP) > 25 mm Hg and pulmonary vascular resistance (PVR) ≥ 3.0 Wood units (WU).

To ensure that the study results are relevant to a more severely affected population, we chose to include patients with moderate to severe pulmonary arterial hypertension (mPAP ≥35 mm Hg) as our study subjects. This selection is based on the fact that patients with moderate to severe pulmonary arterial hypertension are at a higher risk for adverse health outcomes and exhibit more significant physiological changes, thus allowing for a better reflection of disease severity and its impact on patient health. This approach will aid in exploring the clinical characteristics and metabolic changes associated with this condition.

All patients in this study were newly diagnosed with PAH, with no prior history of CHD or a known CHD history but no surgical intervention or treatment. Therefore, all participants had not received PAH medications prior to the study. As the condition progressed, these patients developed symptoms of pulmonary arterial hypertension, but had not yet reached the stage of right heart failure. Relevant hemodynamic data from right heart catheterization will be presented in the associated tables for further analysis.

To assess the adequacy of our sample size, we conducted the following analyses. For the primary metabolomics comparison between isolated CL-RSHD (*n* = 46) and CL-RSHD + PAH (*n* = 27) patients, a post-hoc power analysis was performed using G*Power 3.1.9.4. Assuming a two-tailed t-test with α = 0.05 and a medium-to-large effect size (Cohen’s d = 0.75), the available sample size (*N* = 73) provides 86.25% power, indicating sufficient sensitivity to detect biologically meaningful differences. The total metabolomics cohort (*N* = 86) is consistent with recent exploratory PAH omics studies. For the gut microbiome analysis (*N* = 41), conventional parametric power calculations are not suitable due to the high-dimensional, non-normal nature of 16S sequencing data. We therefore referenced simulation-based guidelines, specifically the micropower framework for PERMANOVA, which indicates that in balanced multi-group comparisons a sample size of ∼30–60 subjects typically provides >90% power to detect moderate effect sizes (ω^2^ ≈ 0.02–0.08) using distance metrics such as UniFrac or Jaccard. Our cohort size (*N* = 41) falls within this empirically supported range and is aligned with common practice in exploratory microbiome research, supporting its ability to detect group-level differences in community structure.

### 2.2 Sample collection and measurements

Stool samples were collected on site at the hospital during inpatient admission or outpatient visits. Samples were self-collected by participants using sterile, preservative-free disposable containers under standardized instructions provided by trained study staff. No stabilizing agents (e.g., RNAlater or OMNIgene•GUT) were used. Approximately 2–5 g of fresh stool was transferred into the container and immediately placed in an insulated cooler at 4 °C. Samples were delivered to the laboratory within 30–60 min of collection. Samples were then rapidly transferred to a −80 °C freezer for long-term storage. The average time from collection to storage at −80 °C was 60–90 min, and all samples were processed within the predefined 2-h window. No environmental exposure occurred during sample processing, and all handling steps were performed using sterile materials to minimize background contamination.

Blood samples were collected within 24 h after stool sample collection, with participants fasting for at least 8 h prior to blood collection. All participants refrained from taking any medications 24 h before sample collection. Fasting venous blood was collected in EDTA-K2 tubes and centrifuged at 4000 rpm for 15 min to separate plasma, which was then stored at −80 °C.

### 2.3 High-throughput 16S ribosomal RNA gene sequencing

Genomic DNA was isolated from fecal samples using the TGuide S96 Magnetic Soil/Stool DNA Kit (Tiangen Biotech Co., Ltd.). Approximately 0.25–0.5 g of each stool sample was homogenized with 0.25 g of sterile grinding beads in lysis buffer by vortexing at maximum speed for 15 min. Subsequently, the mixture was incubated at 70 °C for 15 min to enhance thermal lysis. DNA was purified using the kit’s magnetic bead-based protocol, eluted, and quantified fluorometrically using a Qubit fluorometer (Thermo Fisher Scientific). Samples meeting explicit quality-control thresholds—defined by clear electrophoretic band intensity and a minimum DNA concentration of ≥0.3 ng/μL—were advanced to PCR.

The V3–V4 region of the bacterial 16S rRNA gene was amplified with primers 338F (5′-ACT​CCT​ACG​GGA​GGC​AGC​A-3′) and 806R (5′-GGACTACHVGGGTWTCTAAT-3′). Each sample was processed in a primary PCR reaction for library construction. The first-round PCR (10 µL total volume) contained 2.5–4 ng DNA template, 0.3 µM of each primer, 1× KOD FX Neo Buffer, 200 µM of each dNTP, and 0.2 U/µL KOD FX Neo polymerase (TOYOBO). The thermocycling conditions were: initial denaturation at 95 °C for 5 min; followed by 25 cycles of 95 °C for 30 s, 50 °C for 30 s, and 72 °C for 40 s; with a final extension at 72 °C for 7 min. Amplicons were verified by 1.8% agarose gel electrophoresis. Samples showing weak bands (intensity lower than the 1,500 bp marker) were re-amplified in duplicate or triplicate, and the products were pooled prior to purification. Library construction and paired-end sequencing (2 × 250 bp) were performed on an Illumina NovaSeq 6000 platform at Biomarker Technologies Co., Ltd., following their standard protocols.

### 2.4 Library preparation and sequencing details

Library preparation was performed using a manual two-step PCR-based protocol, rather than a commercial library preparation kit (e.g., Nextera or Illumina 16S), following standardized procedures. Briefly, after initial amplification of the V3–V4 region, amplicons were purified and subjected to a second indexing PCR to incorporate Illumina-compatible dual-index barcodes, allowing multiplexed sequencing. Indexed libraries were quantified, normalized, and pooled at equimolar concentrations. All libraries were sequenced in a single sequencing batch using one flowcell on an Illumina NovaSeq 6000 platform with paired-end reads (2 × 250 bp), thereby minimizing potential batch effects introduced during sequencing. After quality filtering, an average sequencing depth of 69,171 reads per sample was obtained, with a minimum of 48,924 reads per sample. Per-sample sequencing depth information is publicly available in the NCBI Sequence Read Archive under BioProject PRJNA1167013. The use of a single-run, single-flowcell sequencing strategy combined with standardized library preparation and pooling procedures was intended to minimize technical variability and batch effects across samples.

### 2.5 Quality control and contamination management

To ensure data quality and monitor potential contamination, negative controls (DNA extraction blanks and PCR no-template controls) and a positive control (a commercial mock microbial community) were included in every sequencing batch. All controls were processed in parallel with study samples. Negative controls consistently yielded very low read counts, and no abundant taxa overlapping with study samples were detected. The mock community showed the expected taxonomic composition, confirming sequencing accuracy. Potential contaminant taxa were evaluated using prevalence-based filtering, and no taxa required removal. Overall, quality control results indicated minimal background contamination and reliable sequencing performance.

### 2.6 Bioinformatic analysis

A total of 3,059,273 paired-end reads were obtained from the 41 fecal samples. Raw reads were processed using a standardized bioinformatics pipeline. First, paired-end reads were quality-filtered using Trimmomatic (v0.33) with a 50-bp sliding window and an average quality threshold of Q20, followed by primer trimming with Cutadapt (v1.9.1) allowing a maximum error rate of 20%. High-quality reads were then merged, and chimeric sequences were identified and removed using UCHIME (v8.1) within the USEARCH pipeline. After quality control, 2,835,991 high-quality sequences remained, yielding an average depth of 69,171 reads per sample (minimum: 48,924).

Rarefaction curves suggested that the sequencing depth was sufficient to capture most of the microbial diversity across samples (Supplementary Figure S1). Therefore, rarefaction was not applied for downstream analyses to avoid unnecessary data loss.

High-quality sequences were clustered into operational taxonomic units (OTUs) at a 97% sequence similarity threshold using the--usearch_global command in USEARCH (version 2.8.1). This clustering strategy was selected to maintain consistency with established microbial ecology practices, facilitate cross-study comparisons, and ensure stable clustering given the read length and quality characteristics of the sequencing data. Chimera removal was performed using the UCHIME algorithm. Taxonomic assignment was performed using the Naïve Bayes classifier implemented in QIIME2 (version 2020.6), trained on the SILVA 16S rRNA gene reference database (Release 138.1), with a confidence threshold of 70%.

The resulting OTU table was processed for downstream analyses. Given the relatively narrow range of sequencing depth across samples (minimum: 48,924 reads; mean: 69,171 reads), rarefaction was not performed to avoid unnecessary data loss. Instead, OTU tables were normalized using relative abundance for ecological analyses. Alpha diversity indices, including Shannon, Simpson, and Chao1, were calculated on normalized data within QIIME2. Beta diversity was assessed using Bray–Curtis dissimilarity, which is less sensitive to differences in library size when applied to relative abundance data, and visualized by principal coordinate analysis (PCoA).

Group-level differences in microbial community structure were statistically tested using permutational multivariate analysis of variance (PERMANOVA), as implemented in the adonis function of the vegan R package, with 999 permutations. The model included clinical group as the primary explanatory variable. Additional covariates (age, sex, and BMI) were not included because these variables were comparable across groups (Table 1), and inclusion of further covariates could result in model overfitting given the modest sample size.

**TABLE 1:** Hemodynamic, echocardiographic, and arterial blood gas parameters in patients with CL-RSHD and CL-RSHD-associated PAH.

Characteristics		Patients for microbiome analysis (*n* = 41)				Patients for non-targeted metabolome sequencing (*n* = 86)			
		HC (*n* = 9)	CL-RSHD (*n* = 15)	CL-RSHD+PAH (*n* = 17)	*p* value	HC (*n* = 13)	CL-RSHD (*n* = 46)	CL-RSHD+PAH (*n* = 27)	*p* value
Mean age (year)		34.3± 4.9	32.5 ± 3.7	33.7 ± 2.0	*p* > 0.05	34.9 ± 3.4	34.5 ± 14.1	36.1 ± 13.7	*p* > 0.05
Sex	Male, *n* (%)	4, (44.4)	6, (40.0)	7, (41.2)	*p* > 0.05	5, (38.5)	15, (32.6)	11, (40.7)	*p* > 0.05
	Female, *n* (%)	5, (55.6)	9, (60.0)	10, (58.8)	*p* > 0.05	8, (61.5)	31, (67.4)	16, (59.3)	*P* > 0.05
BMI (kg/m^2^)		22.8 ± 2.1	23.5 ± 2.9	23.1 ± 2.5	*p* > 0.05	22.9 ± 2.0	23.4 ± 2.7	23.2 ± 2.4	*p* > 0.05
Smoking (within 3m), n(%)		0	0	0	N/A	0	0	0	N/A
PPI use (within 3m), n(%)		0	0	0	N/A	0	0	0	N/A
Antibiotic use (within 3m), n(%)		0	0	0	N/A	0	0	0	N/A
Alcohol consumption (within 3m), n(%)		0	0	0	N/A	0	0	0	N/A
Complications	Hypertension	0	0	0	N/A	0	0	0	N/A
	Diabetes	0	0	0	N/A	0	0	0	N/A
Laboratory markers	WBC (×10^9^/L)	6.4 (5.6–7.2)	7.5 (6.5–8.5	7.7 (6.7–8.7)	*p* > 0.05	6.3 (5.5–7.1	7.4 (6.4–8.4)	7.6 (6.6–8.6	*p* > 0.05
	RBC (×10^12^/L)	4.5 (4.2–4.8)	4.6 (4.4–5.0)	4.7 (4.4–5.0)	*p* > 0.05	4.5 (4.2–4.8)	4.6 (4.3–4.9)	4.6 (4.3–5.0)	*p* > 0.05
	PLT (×10^9^/L)	235 (200–270)	220 (185–255)	215 (180–250)	*p* > 0.05	240 (205–275)	225 (190–260)	220 (185–255)	*p* > 0.05
	CRP (mg/L)	2.0 (1.5–2.5	2.3 (1.8–2.8)	2.4 (1.9–2.9)	*p* > 0.05	2.1 (1.6–2.6)	2.2 (1.7–2.7)	2.3 (1.8–2.8)	*p* > 0.05
	ALT (U/L)	21 (17–25)	27 (21–33)	28 (22–34)	*p* > 0.05	22 (18–26)	26 (20–32)	27 (21–33)	*p* > 0.05
	Cr (μmol/L)	65 (57–73)	67 (58–76)	69 (60–78)	*p* > 0.05	66 (58–74)	66 (57–75)	68 (59–77)	*p* > 0.05
Diag-nosis	VSD, *n* (%)	N/A	5, (33.33)	N/A	N/A	N/A	14 (30.43)	N/A	N/A
	ASD, *n* (%)	N/A	8 (53.33)	N/A	N/A	N/A	25 (54.35)	N/A	N/A
	PDA, *n* (%)	N/A	2 (13.33)	N/A	N/A	N/A	7 (15.22)	N/A	N/A
	PH due to VSD, *n* (%)	N/A	N/A	6 (35.29)	N/A	N/A	N/A	10 (37.04)	N/A
	PH due to ASD, *n* (%)	N/A	N/A	9 (52.94)	N/A	N/A	N/A	10 (37.04)	N/A
	PH due to PDA, *n* (%)	N/A	N/A	2 (11.76)	N/A	N/A	N/A	7 (25.93)	N/A

All participants with recent exposure to these factors were excluded prior to enrollment; therefore, values are shown for transparency and are not included as covariates in statistical models.

N/A, Not Applicable. HC, healthy controls; CL-RSHD, congenital left-to-right shunt heart disease; PAH, pulmonary arterial hypertension; WBC, white blood cell count; RBC, red blood cell count; PLT, platelet count; CRP, C-reactive protein; ALT, alanine aminotransferase; Cr, creatinine; VSD, ventriculap septal defect; ASD, atrial septal defect; PDA, patent ductus arteriosus.

Differential abundance analysis of microbial taxa was conducted using two complementary statistical approaches to enhance robustness. The primary analysis was performed using DESeq2 on the raw, non-rarefied OTU count data. DESeq2 fits a negative binomial generalized linear model with the design formula ∼ group, comparing healthy controls, CL-RSHD patients, and CL-RSHD + PAH patients. All samples were processed in a single sequencing batch and demographic variables were balanced across groups; therefore, no additional covariates were included. DESeq2’s median-of-ratios method was used for normalization, and Wald tests were applied for statistical inference. Differential taxa were reported as shrunken log_2_ fold changes with corresponding 95% confidence intervals to improve estimate stability. Multiple testing correction was performed across all 7,872 tested OTUs using the Benjamini–Hochberg false discovery rate (FDR), with *q* < 0.05 considered statistically significant.

As a secondary, corroborative analysis, Metastats was applied to identify differentially abundant taxa based on relative abundance data. Metastats performs group-wise comparisons using a t-test framework with permutation-based estimation and applies Benjamini–Hochberg FDR correction (*q* < 0.05). Concordant findings between DESeq2 and Metastats were considered particularly robust, while DESeq2 results served as the primary basis for inference due to its suitability for overdispersed count data and its ability to provide effect size estimates.

### 2.7 Metabolites extraction

Metabolomics analysis was conducted using a Waters Acquity I-Class PLUS UHPLC system coupled with a Waters Xevo G2-XS QTof high-resolution mass spectrometer. The column used was a Waters Acquity UPLC HSS T3 (1.8 µm, 2.1 × 100 mm).

### 2.8 LC-MS/MS analysis

The Waters Xevo G2-XS QTOF mass spectrometer collected primary and secondary data in MSe mode using MassLynx V4.2 software. Dual-channel acquisition occurred simultaneously at low (2V) and high collision energies (10–40V), with a 0.2-s scan frequency. ESI ion source parameters were: capillary voltage 2000V (positive) or −1500V (negative), cone voltage 30V, source temperature 150 °C, desolvation temperature 500 °C, and gas flow rates of 50L/h (backflush) and 800L/h (desolvation).

### 2.9 Data preprocessing and annotation

The raw data obtained from MassLynx V4.2 was processed through Progenesis QI software for peak extraction, alignment, and related procedures. Compound identification was conducted using the METLIN database along with Biomark’s internal library, ensuring theoretical fragment matching and maintaining mass deviation within a 100 ppm threshold.

### 2.10 Data analysis

For microbiome analysis, taxonomic differential abundance testing was performed as detailed in Section 2.6.

In metabolomic profiling, raw peak areas were normalized by probabilistic quotient normalization (PQN) and log-transformed to reduce heteroscedasticity. Technical reproducibility was rigorously monitored using quality control (QC) samples; only metabolite features with a relative standard deviation (RSD) < 20% across QCs were retained for downstream analysis to minimize technical noise. Orthogonal partial least squares-discriminant analysis (OPLS-DA) models were validated via 200 permutation tests (empirical *p* < 0.01), with variable importance in projection (VIP) scores >1.0 used to prioritize discriminatory metabolites. Differential metabolites were defined by fold change (FC) > 1.5, Mann-Whitney U test *p* < 0.05 (FDR-corrected), and VIP >1.0. Functional annotation leveraged KEGG, HMDB, and LIPID MAPS databases, with pathway enrichment assessed via hypergeometric tests (*q* < 0.05).

### 2.11 Statistical analysis

Clinical variables were analyzed following strict adherence to data distribution characteristics. Normality was assessed using the Shapiro-Wilk test (*p* ≥ 0.05 indicating normal distribution). Normally distributed continuous variables were presented as mean ± standard deviation (SD) and analyzed using parametric tests, while non-normally distributed variables were presented as median (interquartile range, IQR) and analyzed using non-parametric methods. Two-group comparisons were conducted using independent samples t-tests for normally distributed data and Mann-Whitney U tests for non-normally distributed data. Multi-group comparisons were performed using one-way ANOVA with post-hoc Tukey HSD (or Bonferroni-adjusted t-tests) for parametric data, or Kruskal-Wallis tests with Dunn’s post-hoc test (Benjamini-Hochberg FDR correction) for non-parametric data.

For omics data (microbial abundances and metabolite concentrations), which inherently exhibit skewed distributions, statistical analyses were performed using non-parametric tests (Mann-Whitney U or Kruskal-Wallis tests). Results were reported as median (IQR), and multiple testing correction was applied using the Benjamini-Hochberg FDR method (*q* < 0.05). Differential microbial taxa were identified using DESeq2 with negative binomial regression models.

Spearman correlation analysis was performed to assess the relationships between microbial taxa and metabolites, with significance defined as |CC| > 0.8 and *p* < 0.05. *P*-values were adjusted for multiple testing using the Benjamini-Hochberg false discovery rate (FDR) procedure.

To evaluate the diagnostic potential of differential metabolites, ROC curve analysis was conducted using the R (3.5.1) package pROC. The area under the curve (AUC) was calculated to assess discriminative power. The optimal threshold was determined using Youden’s index, and the sensitivity and specificity were reported. DeLong’s test was used to compare AUC values between models.

All statistical analyses were performed using IBM SPSS Statistics 26 and R (3.5.1), with significance thresholds set at *p* < 0.05 (two-tailed) for clinical variables and *q* < 0.05 (Benjamini-Hochberg FDR) for high-dimensional omics data.

To ensure the robustness and biological relevance of our findings, stringent quality control and confounder mitigation strategies were implemented throughout the analytical pipeline and study design. More fundamentally, the primary strategy for controlling potential lifestyle and medication confounders (e.g., recent antibiotic/probiotic use, extreme diets, smoking) was proactively addressed through the stringent pre-collection exclusion criteria detailed in Section 2.1. This design-based approach aimed to create analytically comparable groups and reduce spurious associations, thereby strengthening the inference that the observed omics differences are more likely linked to CL-RSHD and PAH status.

## 3 Results

### 3.1 Clinical characteristics of enrolled subjects

A total of 86 participants were enrolled in this study, including 46 patients with CL-RSHD, 27 patients with CL-RSHD + PAH, and 13 healthy controls (HC). Plasma samples were obtained from all participants for untargeted metabolomic analysis, whereas fecal samples were successfully collected from 41 individuals for gut microbiome profiling (15 CL-RSHD, 17 CL-RSHD + PAH, and 9 HC).

As summarized in Table 1, the three groups were well matched in terms of age and sex, with no statistically significant differences observed. In addition, BMI, comorbidities (hypertension and diabetes), medication exposure, and routine laboratory parameters—including complete blood count, inflammatory marker (C-reactive protein), and hepatic and renal function indices—did not differ significantly among groups, indicating comparable baseline clinical status and minimal confounding from systemic inflammation or organ dysfunction.

Hemodynamic, echocardiographic, and arterial blood gas parameters are presented in Table 2. Compared with patients with CL-RSHD alone, patients with CL-RSHD + PAH exhibited significantly impaired gas exchange, characterized by lower arterial partial pressure of oxygen (PO_2_) and oxygen saturation (SaO_2_), accompanied by a mild increase in partial pressure of carbon dioxide (PCO_2_). Consistently, peripheral oxygen saturation measured by pulse oximetry was also reduced in the CL-RSHD + PAH group, reflecting the hypoxemic state associated with pulmonary arterial hypertension. Arterial blood gas analysis was not performed in healthy controls due to its invasive nature.

**TABLE 2:** Hemodynamic, echocardiographic, and arterial blood gas analysis in CL-RSHD + PAH patients included in gut microbiome and plasma metabolome studies.

Indicators		Patients for microbiome analysis			Patients for non-targeted metabolome sequencing		
		CL-RSHD (*n* = 15)	CL-RSHD+PAH (*n* = 17)	*p* value	CL-RSHD (*n* = 46)	CL-RSHD+PAH (*n* = 27)	*p* value
Peripheral oxygen saturation (%)		98.5 ± 1.2	92.3 ± 1.2	<0.001^*^	98.3 ± 1.1	92.4 ± 0.8	<0.001^*^
Arterial blood gas analysis	PCO_2_ (mmHg)	40.5 ± 2.3	42.1 ± 2.2	*p* > 0.05	40.3 ± 3.0	41.5 ± 2.8	*p* > 0.05
	PO_2_ (mmHg)	92.5 ± 4.2	65.0 ± 3.6	<0.001^*^	92.8 ± 4.0	65.9 ± 2.4	<0.001^*^
	SaO_2_ (%)	98.1 ± 0.8	89.3 ± 2.3	<0.001^*^	97.9 ± 0.7	90.0 ± 1.5	<0.001^*^
Echocardiography	LVEDd (mm)	36.8 ± 0.6	36.8 ± 0.6	*p* > 0.05	36.9 ± 1.2	37.2 ± 1.1	*p* > 0.05
	LAS (mm)	34.3 ± 0.7	34.4 ± 0.6	*p* > 0.05	34.2 ± 0.6	34.3 ± 0.4	*p* > 0.05
	RVD (mm)	34.2 ± 1.2	41.7 ± 1.2	<0.001^*^	34.0 ± 1.1	41.6 ± 0.9	<0.001^*^
	RAS (mm)	35.1 ± 1.3	41.1 ± 1.2	<0.001^*^	34.9 ± 1.2	41.3 ± 1.0	<0.001^*^
	LVEF (%)	63.8 ± 1.5	63.3 ± 1.5	*p* > 0.05	64.2 ± 3.5	65.0 ± 3.4	*p* > 0.05
	RVEF (%)	67.5 ± 1.6	68.0 ± 1.5	*p* > 0.05	67.8 ± 1.5	67.9 ± 1.2	*p* > 0.05
Cardiac catheterization	Mean pulmonary artery pressure (mmHg)	18.5 ± 2.1	59.8 ± 14.9	<0.001^*^	18.8 ± 1.9	63.0 ± 3.2	<0.001^*^
	Pulmonary capillary wedge pressure (mmHg)	9.2 ± 1.1	19.1 ± 0.7	<0.001^*^	9.0 ± 1.0	19.5 ± 0.5	<0.001^*^
	Pulmonary vascular resistance (WU)	2.2 ± 0.5	10.3 ± 1.0	<0.001^*^	2.1 ± 0.4	11.6 ± 1.1	<0.001^*^

CL-RSHD, congenital left-to-right shunt heart disease; PAH, pulmonary arterial hypertension; PCO_2_, carbon dioxide; PO_2_, partial pressure of oxygen; SaO_2_, oxygen saturation; LVEDd, left ventricular end-diastolic diameter; LAS, left atrial size; RVD, right ventricular diameter; RAS, right atrial size; LVEF, left ventricular ejection fraction; RVEF, right ventricular ejection fraction. *p < 0.001.

Transthoracic echocardiography revealed right ventricular enlargement in patients with CL-RSHD + PAH; however, right ventricular ejection fraction (RVEF) and left ventricular ejection fraction (LVEF) remained within normal ranges, suggesting preserved systolic function despite right ventricular dilation. Left ventricular end-diastolic diameter (LVEDd) and left atrial size (LAS) were also within normal limits, indicating the absence of overt biventricular dysfunction at this stage of disease (Table 2).

### 3.2 Preserved gut microbiota diversity with disease-specific taxonomic shifts in CL-RSHD + PAH

**FIGURE 2:**
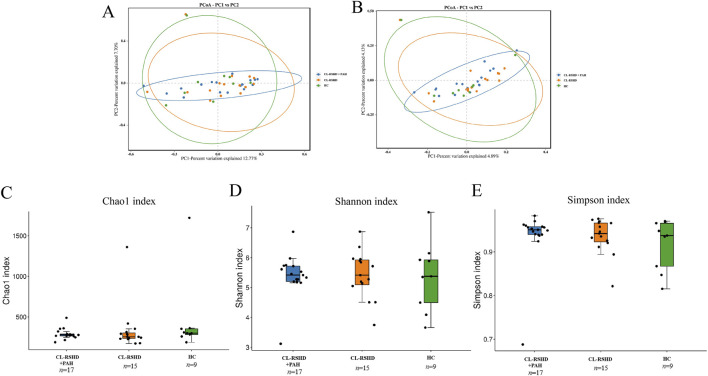
Microbial community composition and α-diversity analysis among study groups. **(A,B)** Principal Coordinate Analysis (PCoA) results: PCoA based on different distance matrices shows the microbial community composition differences among the CL-RSHD+PAH, CL-RSHD, and HC groups. The clustering of sample points represents the similarity in community composition, and the ellipses indicate the 95% confidence interval for each group. **(A)** PCoA based on the Bray-Curtis distance matrix, with PC1 and PC2 explaining 12.77% and 7.70% of the variation, respectively. **(B)** PCoA based on the Weighted UniFrac distance matrix, with PC1 and PC2 explaining 4.89% and 4.13% of the variation, respectively. **(C–E)** α-diversity analysis: **(C)** Chao1 index, representing community richness. **(D)** Shannon index, reflecting microbial diversity. **(E)** Simpson index, representing community evenness. HC,healthy controls; CL-RSHD, congenital left-to-right shunt heart disease; PAH, pulmonary arterial hypertension.

Alpha diversity indices (Chao1, Shannon, Simpson) showed no significant differences in overall microbial richness or evenness among the three groups (all *P* > 0.05, Figures 2C–E), and beta diversity analysis (Bray-Curtis PCoA) revealed overlapping community structures (Figures 2A,B). The robustness of the beta-diversity patterns was further supported by supplementary PCoA visualizations (Supplementary Figure S1). However, significant taxonomic shifts were observed at higher phylogenetic resolutions.

**FIGURE 3:**
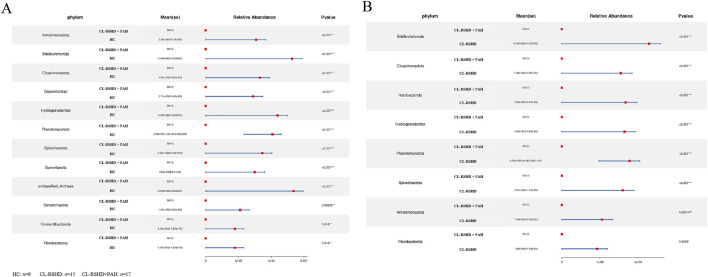
Differential Analysis of Microbial Communities at the Phylum Level. **(A,B)** Comparative analysis of microbial phylum abundance across clinical cohorts. The forest plots illustrate the significantly different phylum abundance between groups, with mean relative abundance (Mean ± SE) and p-values indicated. **(A)** Differences between the CL-RSHD+PAH and HC groups at the phylum level. **(B)** Differences between the CL-RSHD+PAH and CL-RSHD groups at the phylum level. Red squares represent the mean relative abundance, and blue lines indicate confidence intervals. Statistical significance was determined using appropriate tests, with *p < 0.05 and ***p < 0.001. HC, healthy controls; CL-RSHD, congenital left-to-right shunt heart disease; PAH, pulmonary arterial hypertension.

At the phylum level, Metastats analysis identified 12 phyla that were significantly enriched in the CL-RSHD + PAH group compared to the HC group, including Armatimonadota, Bdellovibrionota, Cloacimonadota, Dependentiae, Hydrogenedentes, Planctomycetota, Spirochaetota, Sumerlaeota, unclassified_Archaea, Euryarchaeota, Fermentibacterota, and Fibrobacterota (Figure 3A). Compared to the CL-RSHD group alone, the CL-RSHD + PAH group exhibited increased abundances of eight phyla, namely Bdellovibrionota, Cloacimonadota, Halobacterota, Hydrogenedentes, Planctomycetota, Spirochaetota, Armatimonadota, and Fibrobacterota (Figure 3B). Among these, Halobacterota, Sumerlaeota, and Fermentibacterota were the only phyla that showed significant differences between the HC and CL-RSHD groups (Supplementary Figures S2A). Based on the above results, we further selected gut microbiota specific to CL-RSHD + PAH, i.e., these microbiota exhibited significant differences between the CL-RSHD + PAH group and the healthy group, as well as between the CL-RSHD + PAH group and the CL-RSHD group, but showed no significant differences between the healthy group and the CL-RSHD group. Specifically, Bdellovibrionota, Cloacimonadota, Hydrogenedentes, Planctomycetota, Spirochaetota, Armatimonadota, and Fibrobacterota were identified as potentially unique phylum-level gut microbiota in CL-RSHD + PAH.

**FIGURE 4:**
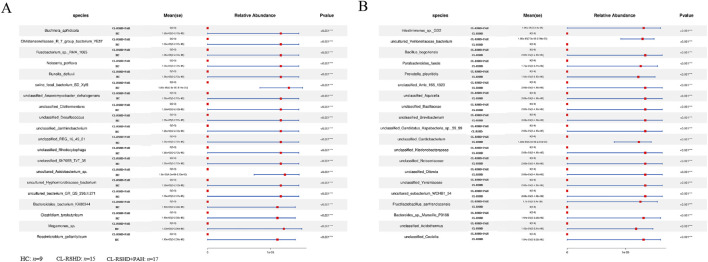
Differentially abundant microbial species between study groups. **(A,B)** Comparison of microbial species relative abundance among groups. The forest plots illustrate the significantly different bacterial species between groups, with mean relative abundance (Mean ± SE) and p-values indicated. **(A)** Differentially abundant species between the CL-RSHD+PAH and HC groups. **(B)** Differentially abundant species between the CL-RSHD+PAH and CL-RSHD groups. Red squares represent the mean relative abundance, and blue lines indicate confidence intervals. Statistical significance was determined using appropriate tests, with ***p < 0.001. HC, healthy controls; CL-RSHD, congenital left-to-right shunt heart disease; PAH, pulmonary arterial hypertension.

Further species-level analysis identified 555 and 350 differentially abundant species in the CL-RSHD + PAH group compared to the HC and CL-RSHD groups, respectively. The top 20 significantly enriched species in each comparison are depicted in Figure 4 and Supplementary Figures S2B. Furthermore, we identified species specific to CL-RSHD + PAH. Several species previously associated with immune-inflammatory responses, including *Alloprevotella tannerae*, *Parabacteroides chongii*, *Prevotella amnii* (phylum Bacteroidetes), *Rothia aeria* (Actinobacteria), *Lachnoclostridium phocaeense* (Firmicutes) and *Neisseria perflava* (Proteobacteria) (Yay et al., 2024; Kim et al., 2018; Wang Q. et al., 2022; Gre et al., 2021; Schmidt et al., 2024; Ren et al., 2023; Jang et al., 2021) were significantly enriched in CL-RSHD + PAH. Additionally, species involved in carbohydrate metabolism, such as *Barnesiella viscericola* (Hu et al., 2019), and those participating in short-chain fatty acid (SCFAs) synthesis, including *Anaerostipes hadrus* (Shinoda et al., 2024), were also identified. Furthermore, we observed a significant decrease in *Clostridium celatum* in the gut microbiota of CL-RSHD + PAH patients. Research indicates its involvement in shaping the intestinal ecosystem alongside other symbiotic bacteria, and its interaction with host intestinal epithelial metabolism. These bacteria produce abundant organic acids and fermentation products, which provide essential carbon and energy sources for other gut microbiota, fostering interdependent relationships (Candeliere et al., 2024).

### 3.3 Quality control and overall metabolic characteristics

**FIGURE 5:**
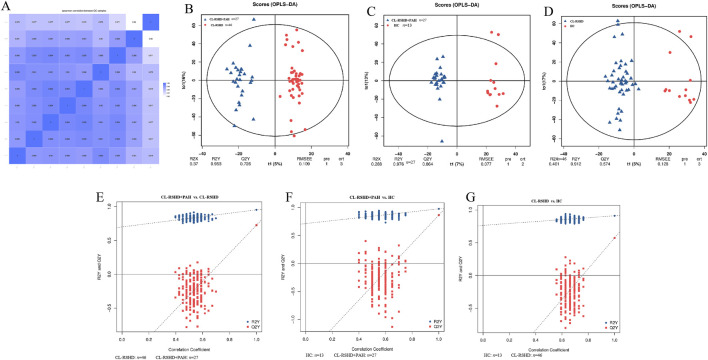
Comparative analysis of metabolomic profiles in pulmonary arterial hypertension and congenital heart disease using OPLS-DA and permutation tests. **(A)** The Spearman correlation heatmap between quality control (QC) samples, indicating high reproducibility and consistency across the metabolomics analysis. **(B)** OPLS-DA score plot comparing the metabolomic profiles between CL-RSHD + PAH and CL-RSHD groups. **(C)** OPLS-DA score plot comparing the metabolomic profiles between CL-RSHD + PAH and HC groups. **(D)** OPLS-DA score plot comparing the metabolomic profiles between CL-RSHD and HC groups. **(E)** Permutation test for the OPLS-DA model comparing CL-RSHD + PAH vs. CL-RSHD, showing model validation and statistical robustness. **(F)** Permutation test for the OPLS-DA model comparing CL-RSHD + PAH vs. HC, confirming model reliability. **(G)** Permutation test for the OPLS-DA model comparing CL-RSHD vs. HC, validating the model’s statistical significance. HC, healthy controls; CL-RSHD, congenital left-to-right shunt heart disease, PAH, pulmonary arterial hypertension.

Untargeted metabolomic profiling was performed on 86 plasma samples. QC analysis demonstrated strong reproducibility and instrument stability, with a minimum correlation coefficient among QC samples >0.75 and at least 60% of total identified peaks exhibiting a coefficient of variation (CV) ≤ 30% (Figure 5A).

To assess metabolic differences between groups, we constructed orthogonal partial least squares discriminant analysis (OPLS-DA) models. These models demonstrated clear separation among the three groups, with high R^2^Y and Q^2^Y values, indicating robust discrimination and predictive performance (Figures 5B–G).

### 3.4 Metabolic Characteristics of Plasma in CL-RSHD+PAH Patients

**FIGURE 6:**
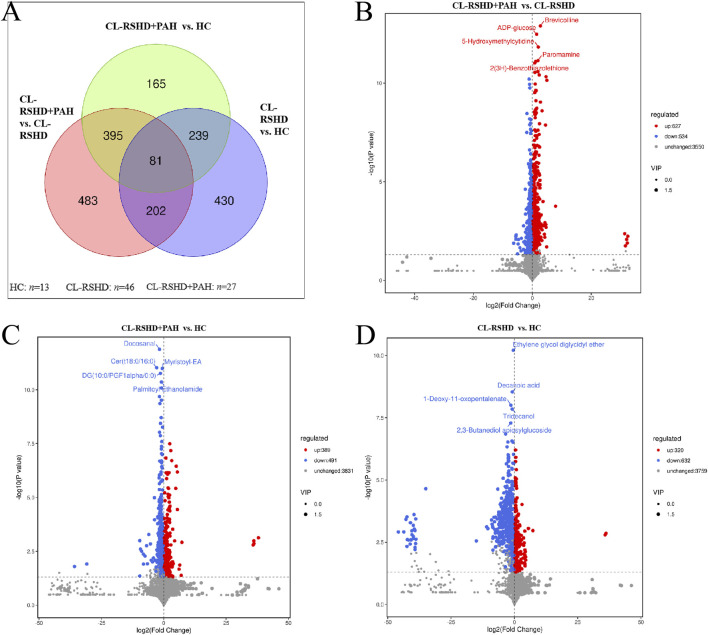
Differential metabolic profiles among HC, CL-RSHD, and patients with CL-RSHD + PAH. **(A)** Venn diagram showing the distribution of differentially expressed metabolites among the three groups: CL-RSHD+ PAH vs. HC, CL-RSHD+ PAH vs. CL-RSHD, and CL-RSHD vs. HC. The numbers in the overlapping regions represent common metabolites, while non-overlapping areas indicate unique metabolites to each comparison group. **(B–D)** Volcano plots illustrating the metabolites significantly altered in the following comparisons: **(B)** CL-RSHD+PAH vs. CL-RSHD, **(C)** CL-RSHD+ PAH vs. HC, and **(D)** CL-RSHD vs. HC. Red dots indicate upregulated metabolites, blue dots represent downregulated metabolites, and gray dots represent unchanged metabolites. The vertical axis shows the -log10(p-value), and the horizontal axis represents the log2(fold change). Metabolites with significant differences are labeled. HC, healthy controls; CL-RSHD, congenital left-to-right shunt heart disease, PAH, pulmonary arterial hypertension.

**FIGURE 7:**
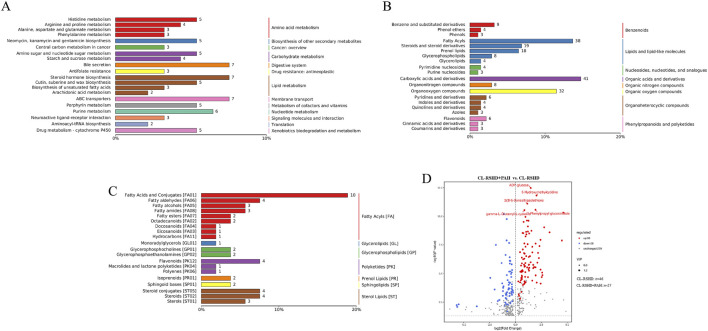
Classification and pathway analysis of metabolites identified in CL-RSHD+ PAH, CL-RSHD, and HC. **(A–C)** Bar charts representing the top 20 annotated metabolites based on detailed classification from KEGG, HMDB, and LipidMAPS databases. **(A)** Metabolic pathways, **(B)** Chemical classification, and **(C)** Lipid subclass distribution. The x-axis shows the percentage of each category, and the numbers at the end of each bar indicate the number of metabolites annotated under each category. **(D)** Volcano plots highlighting the differentially expressed metabolites that are unique to the CL-RSHD+PAH group. These metabolites show significant differences in abundance when comparing CL-RSHD+PAH with CL-RSHD and HC but not between HC and CL-RSHD. Red dots represent upregulated metabolites, blue dots indicate downregulated metabolites, and gray dots represent unchanged metabolites. Significant metabolites are labeled. HC, healthy controls; CL-RSHD, congenital left-to-right shunt heart disease, PAH, pulmonary arterial hypertension.

As shown in Figure 6A, 952 differential metabolites were identified between the healthy group and the CL-RSHD group, 880 differential metabolites between the healthy group and the CL-RSHD+PAH group, and 1161 metabolites between the CL-RSHD group and the CL-RSHD+PAH group. We visually demonstrated the overall trends and statistical significance of differential metabolite levels between each pair of groups using volcano plots, as shown in Figures 6B–D. The identified compounds were grouped, and pathway data were extracted from the KEGG, HMDB, and LIPID MAPS databases. The most thoroughly annotated metabolite categories in each database included chemical structure transformation maps, lipids and related molecules, and fatty acids. From each database, the 20 metabolites with the most comprehensive annotations were selected. A summary bar graph and annotation table were then generated, as shown in Figures 7A–C.

Next, we further filtered for differential metabolites unique to the CL-RSHD+PAH group. These metabolites exhibited significant differences between the CL-RSHD+PAH group and both the healthy control group and the CL-RSHD group, but showed no significant differences between the healthy control group and the CL-RSHD group. A total of 395 metabolites met these criteria, as shown in Supplementary Table S1. Ranked by the p-value of the difference in metabolite levels between the CL-RSHD+PAH and CL-RSHD groups, the top five metabolites were ADP-glucose, 5-Hydroxymethylcytidine (5-hmC), 2(3H)-Benzothiazolethione, 3-Phenylpropyl glucosinolate, and gamma-L-Glutamyl-L-cysteine (γ-Glu-Cys) (Figure 7D).

**FIGURE 8:**
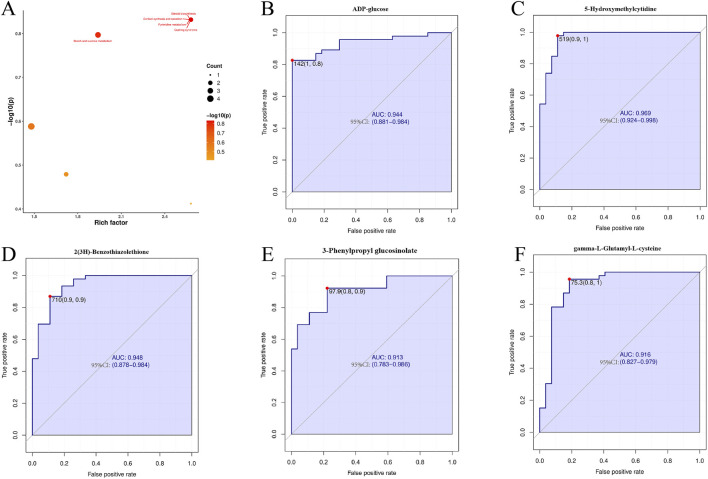
Pathway enrichment and ROC analysis of key metabolites. **(A)** Bubble plot of metabolic pathway enrichment analysis based on rich factor and significance. **(B–F)** Receiver Operating Characteristic (ROC) curves for individual metabolites. The Area Under the Curve (AUC) and its 95% confidence interval (in parentheses) are provided for each biomarker, demonstrating their diagnostic potential.

To further elucidate the biological pathways potentially affected by these differential metabolites, we conducted KEGG pathway enrichment analysis. The results indicated that the altered metabolites were significantly enriched in pathways such as Steroid biosynthesis, Cortisol synthesis and secretion, Pyrimidine metabolism, Cushing syndrome, and Starch and sucrose metabolism (Figure 8A).

### 3.5 Associations between fecal/serum metabolites and gut microbiota

In this study, we performed an integrated analysis of gut microbiota and plasma metabolites in HC, patients with CL-RSHD, and CL-RSHD + PAH to explore their potential roles in the development and progression of PAH. Positive correlations between microbial taxa and metabolites may indicate that a given metabolite promotes the growth of specific microbial species or is produced by them, whereas negative correlations may reflect competitive or inhibitory interactions.

After Benjamini–Hochberg correction, we identified 297 significant associations (|r| > 0.8, FDR-adjusted *P* < 0.05) between 48 differentially abundant plasma metabolites and 257 differentially abundant gut bacterial taxa when comparing the CL-RSHD and CL-RSHD + PAH groups (Supplementary Table S2). Scatterplots for representative microbiome–metabolite correlations, including assessment of potential outlier effects, are provided in Supplementary Figures S3–S5.

As no consistent microbiota–metabolite associations were observed across all three groups (Supplementary Figures S6, S8; Supplementary Tables S2–S4), we further applied a dynamic correlation analysis to identify disease-stage–specific interactions. Specifically, correlations that were not significant in the HC vs. CL-RSHD comparison (FDR-adjusted *P* > 0.05) but became highly significant in both the CL-RSHD vs. CL-RSHD + PAH and HC vs. CL-RSHD + PAH comparisons (|r| > 0.8, FDR-adjusted *P* < 0.05) were considered biologically relevant.

Using this approach, we identified D-erythro-1-(Imidazol-4-yl)glycerol 3-phosphate (D-E1IG3P), a key intermediate in histidine metabolism, which was significantly reduced in CL-RSHD + PAH patients and exhibited a strong positive correlation with the abundance of Lactonifactor (r = 0.82, *P* < 0.01). Histidine metabolism and its derivative histamine have been implicated in the pathogenesis of PAH. Lactonifactor belongs to the phylum Firmicutes, and previous studies have reported a reduction in several Firmicutes-associated taxa in PAH. Together, these findings are consistent with a dysregulated microbiota–metabolite–vascular remodeling axis in CL-RSHD–associated PAH.

To further clarify the potential origins of key differential plasma metabolites identified in CL-RSHD + PAH patients, a database-based traceability analysis was performed by cross-referencing metabolite annotations with public repositories, including HMDB and KEGG. Representative metabolites showing strong disease associations or relevance to the proposed microbiota–metabolite axis were selected for analysis, and the results are summarized in Table 3. As shown in Table 3, several metabolites were annotated as having heterogeneous origins. D-E1IG3P, a key intermediate in histidine metabolism, was mapped to both KEGG and HMDB entries and classified as having a mixed endogenous, microbial, and dietary origin. Similarly, γ-Glu-Cys, an essential intermediate in glutathione metabolism, was annotated as arising from both host endogenous metabolism and microbial pathways.In contrast, 5-hmC was annotated in KEGG as a nucleoside involved in nucleotide metabolism and epigenetic regulation, supporting a predominantly host-endogenous origin. Additionally, ADP-glucose and 2(3H)-benzothiazolethione were annotated as having mixed endogenous and exogenous contributions, whereas 3-phenylpropyl glucosinolate was identified as an exogenous, plant-derived metabolite primarily associated with glucosinolate metabolism (Table 3). Collectively, these results indicate that the differential plasma metabolome in CL-RSHD + PAH patients reflects a complex interplay between host metabolism, gut microbiota-associated pathways, and dietary or environmental inputs, providing a metabolic basis for the proposed regulatory axis.

**TABLE 3:** Traceability analysis and Pathyway annotation of key differential plasma metabolites in CL-RSHD + PAH patients.

Metabolite	HMDB/KEGG ID	Major annotated pathway	Supporting database evidence	Potential origin
D-E1IG3P	HMDB0012208	Histidine metabolism	KEGG& HMDB	Mixed: Endogenous, microbial, and dietary
ADP-glucose	HMDB0006557	Carbohydrate metabolism	KEGG& HMDB	Mixed: Endogenous, microbial, and dietary
​	KEGG C03997	Nucleotide metabolism	KEGG	Host endogenous
2(3H)-benzothiazolethione	HMDB0030524	Xenobiotics/secondary metabolism	HMDB	Mixed: Endogenous & dietary)
3-Phenylpropyl glucosinolate	HMDB0038422	Glucosinolate metabolism	HMDB	Exogenous (Dietary/plant-derived)
γ-Glu-Cys	HMDB0001049	Glutathione metabolism	KEGG& HMDB	Mixed: Host-endogenous & microbial)

HMDB, human metabolome database; KEGG, kyoto encyclopedia of genes and genomes; D-E1IG3P, D-erythro-1-(Imidazol-4-yl)glycerol 3-phosphate; 5-hmC, 5-Hydroxymethylcytidine; γ-Glu-Cys, Gamma-L-Glutamyl-L-cysteine.

### 3.6 Diagnostic potential of plasma metabolites

To evaluate the diagnostic potential of differential metabolites, receiver operating characteristic (ROC) curve analysis was performed. The results demonstrated that metabolites such as ADP-glucose, 5-hmC, 2(3H)-benzothiazolethione, 3-phenylpropyl glucosinolate, and γ-Glu-Cys exhibited excellent diagnostic performance, with AUC values exceeding 0.9. Importantly, 95% confidence intervals (CIs) for all AUC estimates were calculated using the pROC package and are explicitly shown in Figures 8B–F, indicating robust discriminative performance between the experimental and control groups. We further assessed the sensitivity and specificity of each metabolite at the optimal cutoff value, supporting their potential utility as candidate biomarkers for CL-RSHD-associated PAH.

## 4 Discussion

This study utilized 16S rRNA sequencing and untargeted plasma metabolomics to investigate gut microbiota and metabolic alterations in patients with PAH secondary to CL-RSHD. Our findings reveal significant differences in gut microbial composition and plasma metabolomic profiles in PAH patients compared to both healthy controls and CL-RSHD patients without PAH. These results provide new insights into potential disease mechanisms, yet the identification of definitive biomarkers and pathways remains an ongoing challenge requiring further exploration.

The human gut microbiome composition is shaped by numerous factors over the course of life. Studies have shown that, compared to older children and adults, infants have less stable and more variable gut microbiomes (Kashtanova et al., 2016) Generally, under consistent conditions, the composition of the gut microbiome remains relatively stable throughout adulthood (Rodríguez et al., 2015). Therefore, to eliminate the confounding effect of age in this study, we selected adult participants, ensuring that there were no significant age differences among the three groups.

Our data reveal no notable variations in the alpha and beta diversity of the gut microbiota between the CL-RSHD + PAH group and the healthy control and simple CL-RSHD groups. This result aligns with the analysis performed by Ma et al. (Ma et al., 2022). On the lung microbiome in children with congenital heart disease-associated PAH, as well as studies on PAH mouse models by Maria et al. (Callejo et al., 2018), which also did not exhibit significant changes in gut microbiota richness and diversity. However, other clinical studies have reported significantly reduced gut microbiota alpha diversity in PAH patients (Kim et al., 2020; Moutsoglou et al., 2023). The discrepancies in these results may be attributed to insufficient sample sizes and different etiologies. Our study is the first to explore gut microbiota changes in PAH caused solely by CL-RSHD inadults.

Taxonomic shifts at finer phylogenetic levels were evident. Although the Firmicutes/Bacteroidetes (F/B) ratio—a widely studied metric across various pathologies (Petakh et al., 2023; Spychala et al., 2018)—showed no significant alteration in our cohort, taxon-specific changes within these phyla suggest potential mechanistic contributions to PAH pathogenesis. Specifically, the enrichment of *Lachnoclostridium phocaeense* (Firmicutes) and perturbations in Bacteroidetes species, including *Alloprevotella tannerae*, *Parabacteroides chongii*, and *Prevotella amnii*, highlight their disease-modulating roles. These taxa have demonstrated associations with inflammatory and metabolic pathways in prior studies: *Parabacteroides chongii*—first isolated from a patient with peritonitis (Kim et al., 2018)—and Prevotella amnii—closely linked to interleukin (IL)-1β and tumor necrosis factor (TNF)-α fluctuations—may mediate gut microbiota-insomnia interactions through immune modulation (Wang Q. et al., 2022). These findings underscore the importance of analyzing microbial alterations at finer taxonomic resolutions rather than relying solely on phylum-level ratios like F/B, particularly in PAH pathobiology. Additionally, *Rothia aeria* was significantly increased in the CL-RSHD + PAH group, a rare but clinically relevant pathogen implicated in infectious endocarditis (Gre et al., 2021). Inflammatory immune responses, recognized as pivotal drivers of PAH progression and promising therapeutic targets (Wang R. R. et al., 2022), are supported by prior observations of perivascular inflammatory cell infiltration and elevated cytokines/chemokines (e.g., IL-18, TNF-α, IL-6, IL-1β) in PAH patients and animal models (Rabinovitch et al., 2014). However, the causal or compensatory relationship between these microbial changes and pulmonary vascular remodeling remains to be elucidated.

SCFAs are important metabolic products of gut microbiota that can influence cardiovascular diseases through their anti-inflammatory effects (Dinakis et al., 2024; Hong et al., 2024; Ostrowska et al., 2024). Multiple studies have suggested that SCFAs play a potential mechanistic role in regulating PAH. For instance, Karoor et al. demonstrated that the short-chain fatty acid butyrate can mitigate hypoxia-induced PAH by reducing vascular remodeling and inflammation (Karoor et al., 2021). Additionally, María et al. found that acetate levels were significantly decreased in the serum of PAH rats (Callejo et al., 2018). Our data indicate that Anaerostipes, known as an SCFA producer, is significantly reduced in PAH patients. This species also plays an important role in the prevention of type 2 diabetes (Shinoda et al., 2024). Other studies on the gut microbiome of PAH patients have also found a reduction in beneficial SCFA-producing bacteria, such as *Faecalibacterium*, *Butyricimonas*, *Ruminococcaceae*, *Eubacterium*, and *Clostridium* (Kim et al., 2020). Although the specific mechanisms of SCFAs in PAH still need to be explored and validated, our results further support the potential role of SCFAs in the pathogenesis of PAH caused by CL-RSHD.

Metabolomic profiling revealed enriched pathways in steroid biosynthesis, cortisol metabolism, and oxidative stress responses. Notably, 5-hmC—a DNA demethylation marker—generated by the further oxidation of the methylation product 5-methylcytosine (5-mC) in cytosine. Remodeling of 5-mC in DNA is widespread during mammalian development, cellular differentiation, and in the occurrence, progression, and treatment response of cancer (Frye et al., 2016; El-Harakeh et al., 2022). Not only does 5-hmC mark active demethylation, but it is also a relatively stable DNA marker with distinct epigenetic roles. Zhuang et al. found that knockdown of DNA methyltransferase 1 reduces the overall levels of 5-methylcytosine in vascular smooth muscle cells (VSMCs) induced by platelet-derived growth factor, restoring myocardin expression and thereby preventing excessive dedifferentiation, proliferation, migration of VSMCs, and vascular remodeling (Zhuang et al., 2017)—a hallmark of PAH (Tajsic and Morrell, 2011; Morris et al., 2019).

Concurrently, elevated ​3-phenylpropyl glucosinolate and ​γ-Glu-Cys suggest a dual metabolic adaptation. Phenylpropyl glucosinolate is a natural compound belonging to the glucosinolate family. When glucosinolates are hydrolyzed by enzymes upon tissue damage, they produce bioactive isothiocyanates (Morris et al., 2019). Studies have shown that isothiocyanates, such as sulforaphane, activate the Nrf2 pathway to trigger redox signaling, thereby enhancing the body’s endogenous defense mechanisms. This process induces the expression of a series of genes, improving cell survival and enhancing the ability to cope with oxidative stress (Valgimigli et al., 2009; Kwak et al., 2003). Conzatti et al. also demonstrated that sulforaphane can improve right ventricular contractility, mean pulmonary artery pressure, and the redox imbalance in a pulmonary arterial hypertension model (Conzatti et al., 2024).

γ-Glu-Cys, a dipeptide composed of glutamate and cysteine, which serves as a critical intermediate in the synthesis of glutathione (GSH) (Forman et al., 2009). Leopold et al. (Leopold and Loscalzo, 2005)discovered that polymorphisms in antioxidant enzymes, including glutathione peroxidase and glutathione S-transferase, are associated with an increased risk of vascular diseases. This association is linked to their ability to scavenge reactive oxygen species (ROS). In PAH, excessive ROS is a key component of its pathophysiology. A metabolomic analysis by Qin et al. in a rat model of PAH also identified a significant increase in glutathione (Qin et al., 2022). The elevated levels of γ-Glu-Cys, as a crucial intermediate in glutathione synthesis, may represent a cellular response to increased oxidative stress, aiming to enhance antioxidant capacity by boosting glutathione production. However, whether these changes are causal or compensatory requires further investigation.

Notably, given the absence of consistent microbe-metabolite associations across all three groups, we further conducted dynamic correlation analyses to delineate differential interaction networks. Our results revealed a significant reduction in the key histidine metabolism intermediate D-E1IG3P in CL-RSHD + PAH patients, which exhibited strong positive correlation with the abundance of *Lactonifactor* (phylum Firmicutes). Previous studies have demonstrated the dual regulatory role of histidine metabolism and its derivative histamine in PAH pathogenesis. On one hand, diminished D-E1IG3P levels may impair histamine synthesis, thereby attenuating H2 receptor-mediated pulmonary vasodilation (Leary et al., 2018). Conversely, accumulation of its precursor imidazole pyruvate may activate the mTOR signaling pathway, promoting abnormal proliferation of pulmonary arterial smooth muscle cells (Wang et al., 2015). Furthermore, Firmicutes (including *Lactonifactor*) have been shown to participate in host D-E1IG3P biosynthesis through histidine-degrading enzymes or specialized transport systems (Ashniev et al., 2022). The observed depletion of these bacterial taxa may therefore exacerbate D-E1IG3P synthesis deficits. These findings collectively suggest a dysregulated “microbiome-metabolite-vascular remodeling” axis in CL-RSHD-associated PAH, with Firmicutes-histidine metabolic crosstalk emerging as a pivotal regulatory node.

Currently, no potential association between ADP-glucose and 2(3H)-Benzothiazolethione with PAH has been identified. Further investigation is required to determine if these metabolites play any role in the pathogenesis of PAH.

The mechanisms underlying PAH are complex and diverse, involving vascular remodeling, metabolic dysregulation, chronic inflammation, immune dysregulation, and oxidative stress. Although the mechanisms driving PAH secondary to CL-RSHD are not entirely identical to those of other PAH forms (such as idiopathic PAH and chronic thromboembolic PAH), and while the changes in gut microbiota and metabolites also vary, these alterations may similarly influence PAH development by affecting immune-inflammatory factor expression, oxidative stress processes, VSMC proliferation and migration, or by acting through established pathways like the ROS pathway. On the other hand, hypoxia is a driving factor in PAH and can induce various physiological changes, including systemic metabolic alterations and inflammatory responses (Ghofrani et al., 2006). In this study, both peripheral oxygen saturation and arterial blood gas analysis indicated significant hypoxemia in the CL-RSHD + PAH group. Studies have shown that chronic hypoxia leads to inflammation, oxidative stress, metabolic dysfunction, apoptosis, and gastrointestinal barrier damage. It also disrupts the composition and function of the gut microbiota, resulting in altered microbial diversity and a reduction in beneficial SCFA-producing bacteria (Cao et al., 2024), which further exacerbates systemic inflammation and vascular remodeling. These findings align with the gut microbiota alterations observed in PAH patients in our study. In addition to affecting the gut microbiota, hypoxia can also alter the plasma metabolomic profile. Hypoxia-induced oxidative stress and metabolic dysregulation may influence the levels of metabolites involved in energy metabolism, lipid synthesis, and oxidative pathways. For instance, increased ROS production under hypoxic conditions may trigger compensatory metabolic shifts, leading to elevated levels of intermediates in the γ-Glu-Cys and glutathione synthesis pathways (Kwon et al., 2019). These metabolic changes may represent adaptive responses to increased oxidative stress, but they could also promote disease progression by contributing to vascular dysfunction and inflammation. Further investigation into the complex interplay between hypoxia, gut microbiota, and plasma metabolites in CL-RSHD secondary to PAH may not only provide insights into the pathogenesis but also reveal novel therapeutic targets to mitigate disease progression.

Importantly, our database-based traceability analysis (Table 3) provides critical context for interpreting the observed microbiota–metabolite associations. The annotated origins of the key differential metabolites suggest that the plasma metabolic signature in CL-RSHD + PAH does not arise from a single exclusive source but rather reflects dynamic crosstalk among host metabolism, gut microbial activity, and exogenous inputs.For example, D-E1IG3P was identified as a histidine metabolism intermediate with mixed endogenous, microbial, and dietary origins. Given that histidine biosynthesis pathways are highly conserved across both host cells and various gut microorganisms (particularly Firmicutes), the strong positive correlation between D-E1IG3P and *Lactonifactor* abundance is biologically plausible. While this does not imply exclusive microbial biosynthesis, it likely reflects coordinated regulation within a shared host-microbe metabolic network.In contrast, 5-hmC was annotated as a host-endogenous metabolite involved in epigenetic regulation. Its significant alteration in CL-RSHD + PAH patients thus highlights a host-specific epigenetic response to disease-induced stress, independent of direct microbial production. Furthermore, the mixed origin of γ-Glu-Cys aligns with its central role in glutathione biosynthesis, a pathway sensitive to oxidative stress mediated by both host defense and microbial metabolism. Collectively, these findings support the conceptualization of the microbiota–metabolite–PAH axis as a putative regulatory network. While our traceability analysis strengthens the biological plausibility of these associations, future studies incorporating isotope tracing or metagenomic functional profiling will be essential to definitively establish the causal directionality and precise source contributions.

Since our study subjects were all newly diagnosed PAH patients who had not undergone surgical treatment for congenital heart disease, the study results were not influenced by postoperative complications or long-term medication effects. As PAH progresses, right heart failure may have a more significant impact on the gut microbiota; however, in this study, although right ventricular enlargement was observed in patients with PAH, both the RVEF and LVEF remained within normal ranges, indicating that these patients had not progressed to right heart failure.

This study has certain limitations. First, while our sample size met the requirements for detecting medium effect sizes in this exploratory study, the findings need validation in larger, multi-center cohorts. Second, all participants were recruited from a single tertiary care center, which may limit generalizability to other healthcare settings or ethnic populations. In addition, although rarefaction curves indicated sufficient sequencing depth across all samples, α- and β-diversity estimates may still be influenced by sequencing depth variation and the choice of normalization strategy. In this study, rarefaction was not applied in order to avoid unnecessary data loss, and relative-abundance normalization was used for ecological analyses. While this approach is widely accepted, particularly when sequencing depth variation is limited, it may affect the sensitivity of diversity metrics, especially for low-abundance taxa. Therefore, the observed diversity patterns should be interpreted with appropriate caution. These diversity analyses primarily provide supportive ecological context rather than serving as the primary basis for inference. Finally, further research is needed to validate the associations between differentially abundant microbiota, plasma metabolites, and the pathophysiology of PAH.

Furthermore, our study has additional methodological considerations that warrant discussion. First, regarding potential microbial contamination, although stringent laboratory protocols were followed and negative controls were included during DNA extraction and amplification, the influence of low-level background DNA from extraction kits and reagents (“kitome”) cannot be entirely excluded, particularly for low-abundance taxa. While no systematic contamination signals were detected and the impact on identifying robust, disease-associated taxonomic differences between clinical groups is likely limited, this remains a methodological consideration for the precise estimation of microbial abundances. Second, while our primary bioinformatic analysis and reported findings are based on OTUs clustered at 97% sequence similarity, we acknowledge that more recent ASV-based methods can offer finer taxonomic resolution and improved reproducibility by avoiding arbitrary clustering thresholds. Accordingly, our OTU-based approach is well suited for capturing robust, group-level ecological differences, but some subtler, strain-level variations may not have been fully resolved. Third, although participants with extreme dietary habits were excluded through stringent enrollment criteria, the absence of quantitatively detailed dietary records limits our ability to fully adjust for or explore the nuanced role of diet as a potential confounder or effect modifier of gut microbiota composition. This limitation is shared by many exploratory clinical microbiome studies and highlights the importance of integrating comprehensive dietary assessments in future work. Finally, partial sample loss for fecal collection resulted in a smaller microbiome cohort compared with the metabolomics cohort, introducing the potential for selection bias. However, participants who provided fecal samples remained representative with respect to key demographic, clinical, and metabolic characteristics (Table 1). Collectively, these factors may affect the precision of microbial abundance estimates and the generalizability of specific microbiota–metabolite associations to other populations with different genetic backgrounds, dietary patterns, or laboratory protocols. Nevertheless, the consistency of our findings with established biological pathways and prior studies supports the validity of the core conclusions regarding systemic microbial and metabolic dysregulation in CL-RSHD with PAH.

## 5 Conclusion

CL-RSHD-associated PAH is characterized by gut microbial restructuring and metabolic reprogramming linked to immune-inflammatory activation, oxidative stress, and vascular remodeling. The Firmicutes-histidine metabolism axis emerges as a potential therapeutic target. Despite limitations in sample size and single-center design, this study provides foundational insights into microbial-metabolic drivers of PAH, warranting validation in larger cohorts.

## Data Availability

The datasets generated and/or analyzed during the current study are publicly available in the NCBI repository’s BioProject database, with the accession number PRJNA1167013.
