# Supplementary figures and images for: Alterations in gut microbiota and plasma metabolites in pulmonary arterial hypertension secondary to congenital left-to-right shunt heart disease: potential mechanisms and biomarkers
Source: Front Genet. 2026 Feb 6;17:1699787. doi: 10.3389/fgene.2026.1699787 (PMC12921412; doi:10.3389/fgene.2026.1699787)

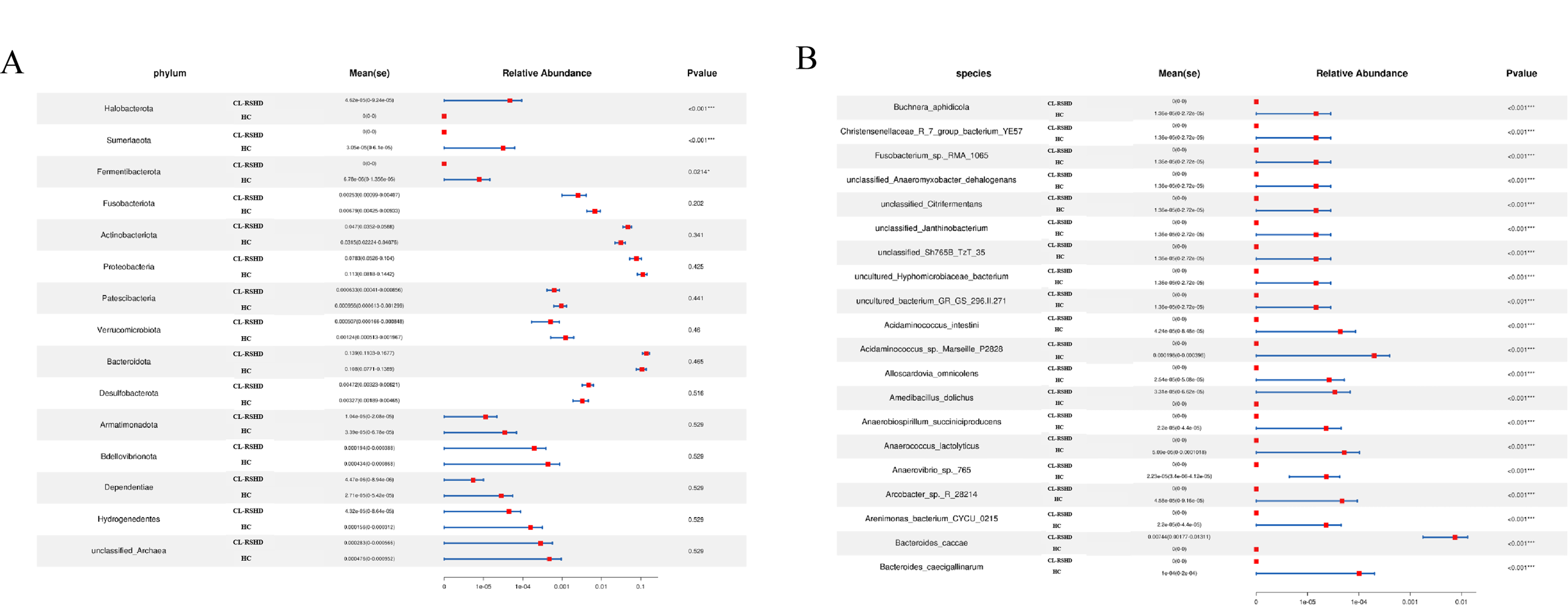

Supplement: Supplementary file 6 [file Image2.jpeg]

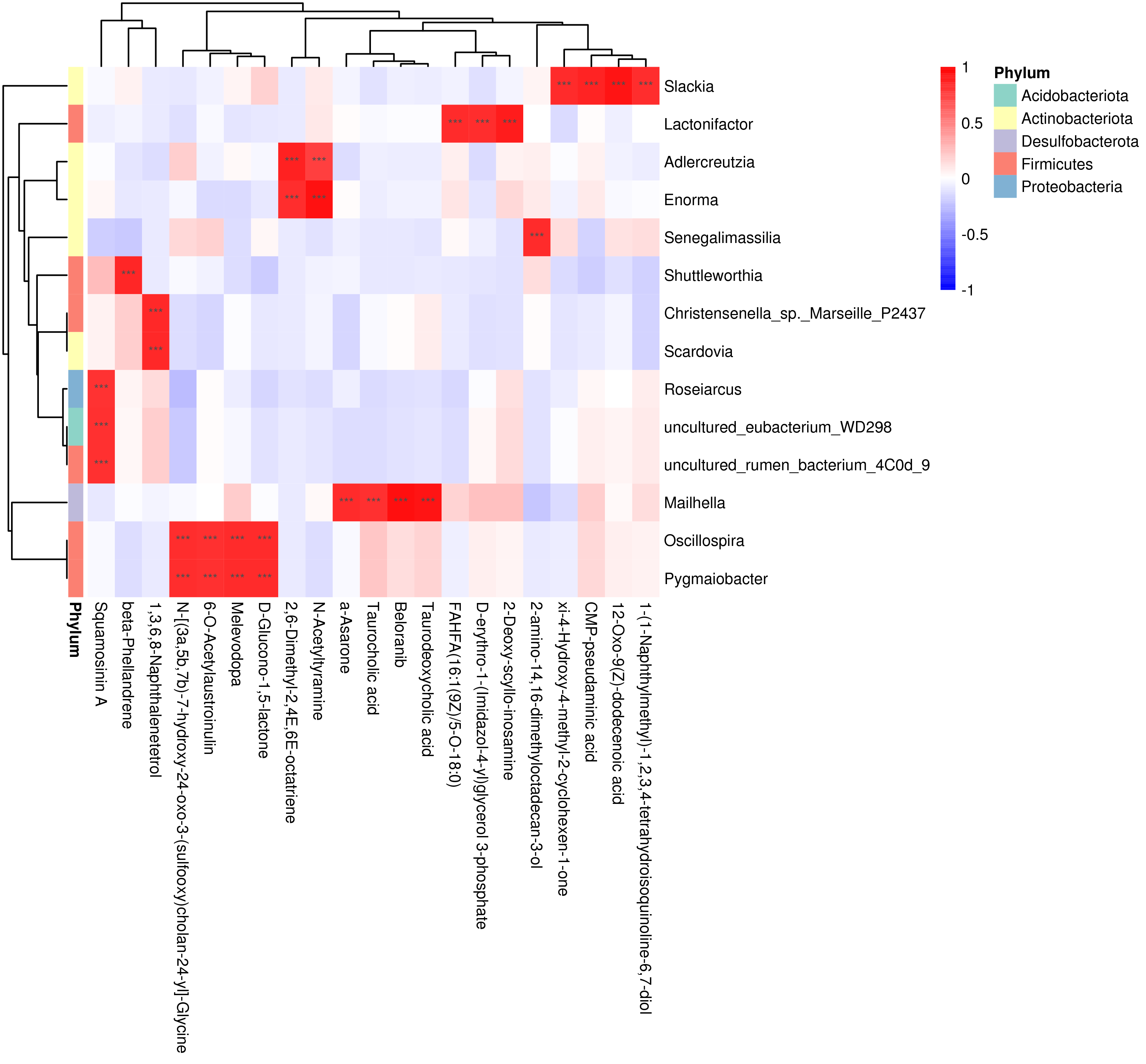

Supplement: Supplementary file 9 [file Image7.png]

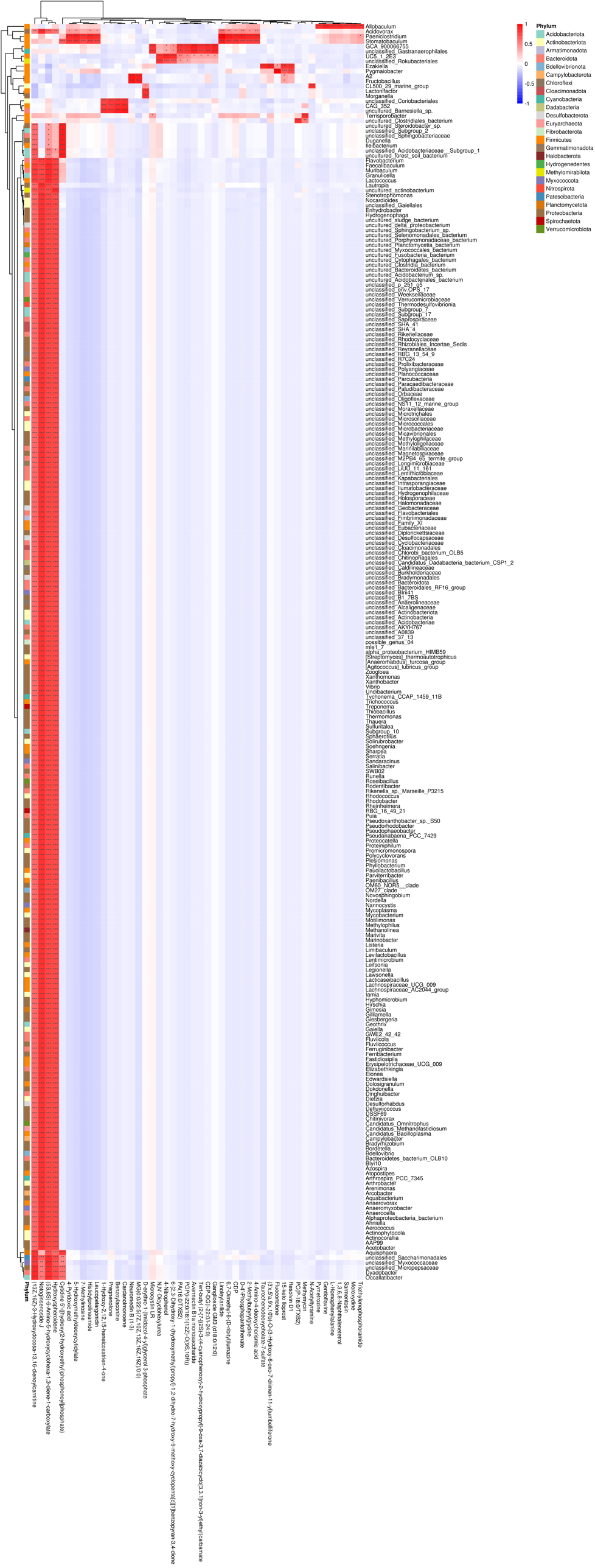

Supplement: Supplementary file 12 [file Image6.jpeg]
